# Supplementary material for: Synthesis of Di-Block Copolymers Poly (Propylene oxide)-block-Poly (9-(2,3-epoxypropyl) Carbazole) via Sequential Addition of Monomers in One Pot
Source: Polymers (Basel). 2021 Feb 28;13(5):763. doi: 10.3390/polym13050763 (PMC7957571; doi:10.3390/polym13050763)
Supplement: Supplementary file 1 [file polymers-13-00763-s001.pdf]

Supplementary Materials

Synthesis of Di-Block Copolymers Poly (propylene oxide)-block-Poly (9-(2,3-epoxypropyl) Carbazole) via Sequential Addition of Monomers in One Pot

Lorella Izzo[1], Paola Lisa[2], Olga Sacco[2], and Stefania Pragliola[2,*]

[1] Università degli Studi dell'Insubria, Dipartimento di Biotecnologie e Scienze della Vita, Via J.H. Dunant, 3 – 21100 Varese, Italy; lorella.izzo@uninsubria.it (L.I.)
[2] Università di Salerno, Dipartimento di Chimica e Biologia, and INSTM Research Unit, Via Giovanni Paolo II 132, I-84084 Fisciano, SA, Italy; lisa91paola@gmail.com (P.L.); osacco@unisa.it (O.S.)
* Correspondence: spragliola@unisa.it (S.P.); Tel.: ++39 089 969580

600 MHz 2D DOSY NMR of PPO-*b*-PEPK
Diffusion Analysis

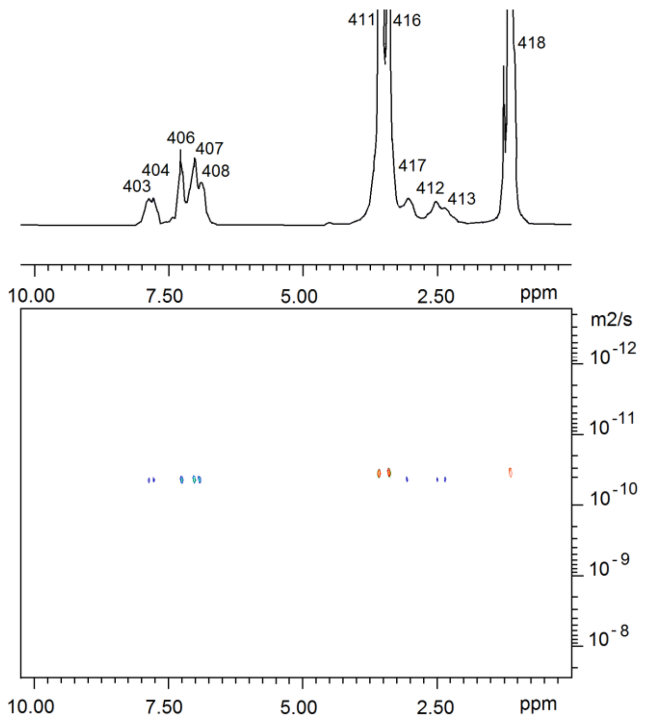

Fitted function:	f (x) = Io * exp (-D * x^2 * gamma^2 * littleDelta^2 (bigDelta-littleDelta/3)* 10^4
used gamma:	26752 rad/(s*Gauss)
used little delta:	0.0056000 s
used big delta:	0.14990 s
used gradient strength:	variable
Random error estimation of data:	RMS per spectrum (or trace/plane)
Systematic error estimation of data:	worst case per peak scenario
Fit parameter Error estimation method:	from fit using arbitray y uncertainties
Confidence level:	95%
Used peaks:	automatically picked peaks
Used integrals:	peak intensities
Used Gradient strength:	all values (including replicates) used

Peak name	F2 [ppm]	D [m2/s]	error
403	7.873	4.33e-11	1.816e-12
404	7.769	4.03e-11	1.488e-12
406	7.261	4.24e-11	2.354e-12
407	7.021	4.03e-11	1.480e-12
408	6.903	3.99e-11	1.472e-12
411	3.052	4.09e-11	1.517e-12
412	2.522	4.24e-11	2.069e-12
413	2.424	4.02e-11	2.126e-12
416	3.563	3.42e-11	4.387e-13
417	3.409	3.42e-11	4.373e-13
418	1.154	3.42e-11	4.302e-13
